# Drowsiness Classification in Young Drivers Based on Facial Near-Infrared Images Using a Convolutional Neural Network: A Pilot Study

**DOI:** 10.3390/s25216755

**Published:** 2025-11-04

**Authors:** Ayaka Nomura, Atsushi Yoshida, Takumi Torii, Kent Nagumo, Kosuke Oiwa, Akio Nozawa

**Affiliations:** 1 Department of Electrical Engineering and Electronics, Aoyama Gakuin University, Sagamihara 252-5258, Japan; 2 Department of Information and Management Systems Engineering, Nagaoka University of Technology, Niigata 940-2188, Japan

**Keywords:** drowsiness classification, facial near-infrared image, convolutional neural network, Grad-CAM

## Abstract

Drowsy driving is a major cause of traffic accidents worldwide, and its early detection remains essential for road safety. Conventional driver monitoring systems (DMS) primarily rely on behavioral indicators such as eye closure, gaze, or head pose, which typically appear only after a significant decline in alertness. This study explores the potential of facial near-infrared (NIR) imaging as a hypothetical physiological indicator of drowsiness. Because NIR light penetrates more deeply into biological tissue than visible light, it may capture subtle variations in blood flow and oxygenation near superficial vessels. Based on this hypothesis, we conducted a pilot feasibility study involving young adult participants to investigate whether drowsiness levels could be estimated from single-frame NIR facial images acquired at 940 nm—a wavelength already used in commercial DMS and suitable for both physiological sensitivity and practical feasibility. A convolutional neural network (CNN) was trained to classify multiple levels of drowsiness, and Gradient-weighted Class Activation Mapping (Grad-CAM) was applied to interpret the discriminative regions. The results showed that classification based on 940 nm NIR images is feasible, achieving an optimal accuracy of approximately 90% under the binary classification scheme (Pattern A). Grad-CAM revealed that regions around the nasal dorsum contributed to this, consistent with known physiological signs of drowsiness. These findings support the feasibility of NIR-based drowsiness classification in young drivers and provide a foundation for future studies with larger and more diverse populations.

## 1. Introduction

In the 21st century, automotive safety technologies have made remarkable progress. The widespread adoption of advanced driver assistance systems (ADAS), such as automatic emergency braking (AEB) and lane departure warning (LDW), has significantly improved the ability of vehicles to avoid accidents. Nevertheless, the majority of traffic accidents are still caused by human error, with decreased driver attention, drowsiness, and cognitive failures remaining major challenges.

According to the World Health Organization (WHO) Global Status Report on Road Safety 2023, approximately 1.19 million people die each year in road traffic crashes worldwide. The report highlights that this burden is particularly pronounced in low- and middle-income countries, and that road traffic injuries are the leading cause of death globally among young people aged 5 to 29 years [1]. In addition, according to the National Highway Traffic Safety Administration (NHTSA), driver misjudgment and inattention are major contributing factors in road crashes, with drowsy driving alone estimated to be involved in approximately 91,000 police-reported crashes in the United States in 2017 [2]. In addition to fatigue and drowsiness, distractions from smartphone use, the influence of alcohol and drugs, and age-related factors all compromise driver concentration. As a result, safety risks have emerged that cannot be addressed by vehicle control technologies alone.

Against this backdrop, driver monitoring systems (DMS) have attracted increasing attention in recent years. DMS monitor the driver’s state, such as drowsiness and inattention, using in-vehicle cameras and provides timely warnings. In Europe, DMS installation has been mandatory in new vehicles since 2022 [3], and similar regulations are being accelerated in Japan [4] and other countries [5,6,7]. Importantly, DMS are not merely alerting systems but are also recognized as a key element of human-centered design in the era of automated driving. By accurately assessing driver states, they enable safe authority transfer between the driver and the automated system, thereby supporting the transition toward higher levels of automation (L3–L4) [8,9].

Currently, most commercial DMS rely on behavioral indicators such as blink rate, eyelid closure, gaze direction, head pose, and body posture [10,11,12,13,14,15,16,17,18,19]. Previous studies on driver monitoring have often been conducted with a small number of participants under controlled laboratory conditions to verify methodological feasibility [11,18,20]. These examples indicate that small-sample pilot studies are widely accepted in the early phase of driver state estimation research, providing valuable insights before large-scale validation. However, these signals tend to manifest only after a significant decline in alertness, limiting their usefulness for early drowsiness detection [21]. Moreover, behavioral features are only external manifestations and do not directly reflect the driver’s physiological state [22]. This gap underscores the need for indices that more directly capture physiological changes related to drowsiness.

Near-infrared (NIR) imaging is considered a promising approach for exploring physiological correlates of drowsiness. The NIR spectrum lies between visible and far-infrared light and contains a wavelength band with relatively high penetration into biological tissues, often referred to as the biological window. The primary absorbers in biological tissue are hemoglobin and water: light below 700 nm is strongly absorbed by hemoglobin, while absorption by water increases markedly above 1200 nm. Consequently, the 700–1200 nm band minimizes absorption by both and allows deeper light penetration than visible light, suggesting that NIR reflectance may contain information related to subsurface blood perfusion and other physiological changes [23,24]. Based on this optical characteristic, the present study investigates whether facial NIR images could serve as potential physiological indicators of drowsiness.

Previous studies have shown the feasibility of using NIR imaging to estimate physiological or psychological states. For example, blood pressure estimation from facial NIR images has been demonstrated [25,26]. In addition, facial NIR-based methods for noninvasive glucose estimation have also been reported [27,28]. These findings suggest that NIR imaging can provide information beyond behavioral features and may reflect underlying physiological processes. Based on this perspective, we hypothesize that facial NIR images can be interpreted as a physiological indicator, capturing skin tone variations associated with drowsiness.

In particular, this study focuses on the 940 nm wavelength, which is already widely used in commercial driver monitoring cameras [29,30,31,32,33]. By utilizing the same spectral band as current systems, the proposed approach aims to reduce barriers to real-world implementation while exploring the feasibility of early-stage drowsiness detection. This work is therefore positioned as a pilot study for NIR-based drowsiness monitoring. To the best of our knowledge, only very limited prior research has attempted to use facial NIR images as physiological indicators of drowsiness. For instance, our group has previously reported a preliminary approach based on sparse coding [20].

To investigate this hypothesis, we conducted a pilot experiment in which facial NIR images were acquired from multiple participants under controlled driving-simulation conditions. Images were captured at 940 nm using a commercially compatible camera system. Drowsiness levels were independently annotated based on external facial expression evaluation, serving as ground-truth labels. The constructed dataset was then used to train a convolutional neural network (CNN) [34,35] for multi-level drowsiness classification because CNNs have become the state-of-the-art approach in image recognition tasks. Furthermore, Gradient-weighted Class Activation Mapping (Grad-CAM) [36] was applied to visualize and interpret the regions of the face that contributed most to the CNN’s decisions. This combined approach allowed us not only to evaluate the feasibility of NIR-based early drowsiness detection but also to gain insights into the physiological relevance of the learned representations.

The contributions of this study are summarized as follows:

1. Novel interpretation of NIR facial images: This study proposes to treat 940 nm NIR images as physiological indicators rather than behavioral cues, highlighting their potential to capture drowsiness-related changes in facial skin tone.
2. Demonstration of CNN-based drowsiness classification: By applying a CNN to facial NIR images acquired at 940 nm, the feasibility of multi-level drowsiness classification is demonstrated.
3. Pilot study toward practical applications: Since the 940 nm wavelength is already employed in commercial driver monitoring systems, this approach offers a practical pathway toward real-world implementation.

The remainder of this paper is organized as follows. Section 2 reviews previous studies on driver monitoring systems and identifies key research gaps motivating this work. Section 3 describes the data acquisition protocol, ground-truth labeling procedure, dataset construction, CNN model, evaluation metrics, and Grad-CAM visualization method. Section 4 presents the classification results. Section 5 discusses the physiological interpretation of the CNN’s learned representations. Finally, Section 6 concludes the paper and outlines directions for future research.

This work was conducted as a pilot study, meaning a preliminary investigation with a small number of participants under controlled conditions to test the feasibility of a new methodology before large-scale validation. The primary goal was to examine whether near-infrared (NIR) facial images could serve as physiological indicators of drowsiness when analyzed with a CNN model. The experimental setup was designed to approximate in-vehicle monitoring environments, thereby providing both feasibility evidence and a basis for future extensions under real driving conditions. In this study, we specifically focused on young adult drivers, as this group represents the majority of active drivers and allows the examination of drowsiness-related physiological responses under relatively uniform conditions. By limiting the participant demographic to young adults, potential variability due to age-related physiological and behavioral differences—such as skin reflectance, vascular reactivity, and facial muscle tone—was minimized. This design enables a clearer evaluation of the feasibility of near-infrared (NIR)-based drowsiness detection before extending the framework to older or more diverse populations in future studies.

## 2. Related Work

Driver monitoring systems (DMS) have been studied using a wide variety of behavioral and physiological indicators to estimate drowsiness or attention decline. Early systems relied primarily on behavioral cues such as eye blinks, eyelid closure (PERCLOS), gaze direction, head pose, and body posture. More recent approaches have introduced physiological sensors such as EEG, ECG/HRV, respiratory signals, or cerebral blood flow (fNIRS). Table 1 summarizes representative studies, their methodologies, and reported performance.

Most of the above studies rely on behavioral cues or sequential physiological signals acquired over several seconds. Consequently, they generally detect drowsiness only when the driver’s arousal level has already dropped markedly—for example, when eyelid closure, head droop, or autonomic fluctuations become prominent. In addition, many methods require wearable sensors (EEG, ECG, fNIRS) or are sensitive to environmental conditions such as illumination and driver posture.

### 2.1. Research Gap

While behavioral and physiological indicators have proven useful, they commonly depend on temporal feature accumulation, which limits responsiveness for real-time applications. Detection of mild or pre-drowsiness states remains particularly challenging.

### 2.2. Present Approach

To overcome these limitations, the present study focuses on spatial physiological information embedded in a single near-infrared (NIR) facial image. By employing a convolutional neural network (CNN) without time series input, the proposed method enables instantaneous, non-contact estimation of driver drowsiness levels. This non-sequential, single-frame approach mitigates sensor attachment issues and environmental sensitivity, and has the potential to detect subtle physiological changes preceding overt behavioral signs of drowsiness.

## 3. Materials and Methods

In this study, facial near-infrared images corresponding to all drowsiness levels were collected through experiments for drowsiness classification using CNNs. In addition, the ground-truth drowsiness levels were obtained through facial expression assessments conducted by evaluators other than the participants. Further details are provided in the following sections.

### 3.1. Facial NIR Imaging System and Experimental Setup

This subsection describes the imaging system and experimental setup used to acquire facial near-infrared (NIR) images under drowsiness-inducing conditions.

#### 3.1.1. Experimental Systems

**Figure 1:**
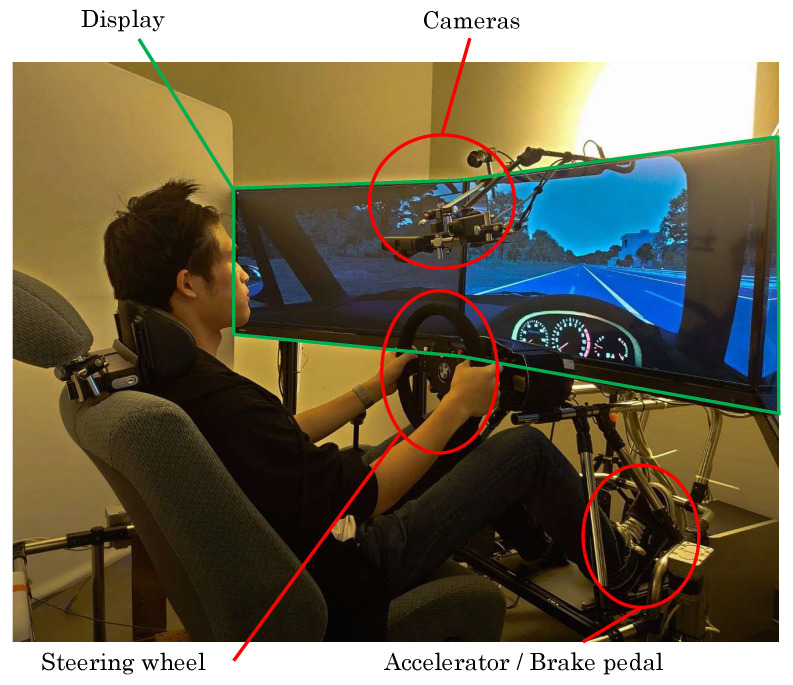
The experimental setup.

The hardware configuration of the NIR imaging system and driving simulator is summarized below. The experimental setup is shown in Figure 1.

Facial near-infrared (NIR) images were captured using a NIR camera (Genie Nano M1280 NIR, Teledyne DALSA, Waterloo, Canada). The camera provides a resolution of 1280×1024 pixels, with a spectral sensitivity of approximately 400–1000 nm, and the image acquisition rate was set to 1 fps. During image acquisition, the subject’s face was illuminated with LED light at a wavelength of 940 nm. A long-pass filter with a cut-on wavelength of 780 nm (RG780, SCHOTT, Mainz, Germany) was placed in front of the NIR camera to block visible light. In addition, a polarizer (SPFN-30C-26, Sigma Koki, Tokyo, Japan) was installed in front of both the LED and the NIR camera to measure scattered light from the skin.

#### 3.1.2. Procedure and Conditions

The experimental procedure and pre-experimental conditions were designed to ensure consistency across participants and to effectively induce drowsiness.

In this experiment, a driving task using a driving simulator was assigned to the participants in order to record facial near-infrared (NIR) images under drowsiness-inducing conditions. The participants were 10 healthy adults (male and female) who held a standard driver’s license (mean age: 22.7 ± 0.9 years). Prior to the experiment, they were instructed to sleep at least 6 h, refrain from food and drink (except water), smoking, sleeping, and exercise for 2 h before the experiment, and participate without makeup, sunscreen, or other facial coverings. During the experiment, subjects were required to wear no visual aids other than eyeglasses or contact lenses. Two experimenters were present in the same room to monitor the physiological measurement conditions. To allow acclimatization to room temperature, the experiment was initiated at least 30 min after the subjects entered the room. Furthermore, to allow participants to adapt to the steering, accelerator, and brake operations of the driving simulator, a 15 min practice session was conducted. Each participant completed a single session composed of three consecutive periods, as illustrated in Table 2:

Thus, the total duration per participant was 46 min. Facial NIR images were recorded throughout the session according to this timeline. During the eyes-open resting period, a white cross was presented on a black background on the liquid crystal display in front of the participant, and the subjects were instructed via voice guidance to fixate on the cross. During the driving period, participants were instructed to maintain a constant driving speed of 80 km/h using the driving simulator. To enhance the drowsiness-inducing effect, the driving posture and steering style were left to the discretion of the subjects.

**Figure 2:**
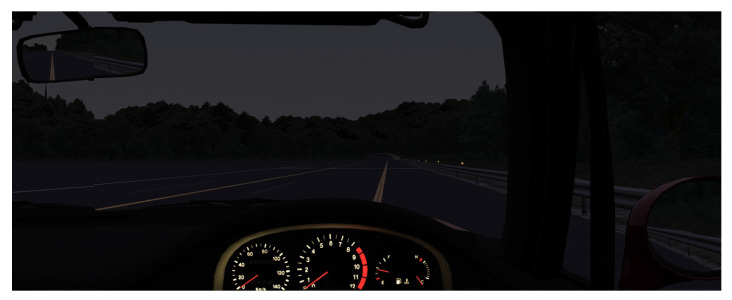
The driving screen.

The driving simulator consisted of an accelerator and brake pedal set, a steering wheel controller (FANATEC ClubSport Wheel, Endor AG, Landshut, Germany), and three LCD monitors (40S21, Toshiba Visual Solutions Corporation, Tokyo, Japan). The simulation software used was Assetto Corsa (ver. 1.0, Kunos Simulazioni S.r.l., Rome, Italy). The vehicle model was based on the Suzuki Cappuccino (Suzuki Motor Corporation, Hamamatsu, Japan), a popular Japanese compact car, and the driving course was a flat, monotonous oval track with a total length of approximately 5500 m (Figure 2).

**Figure 3:**
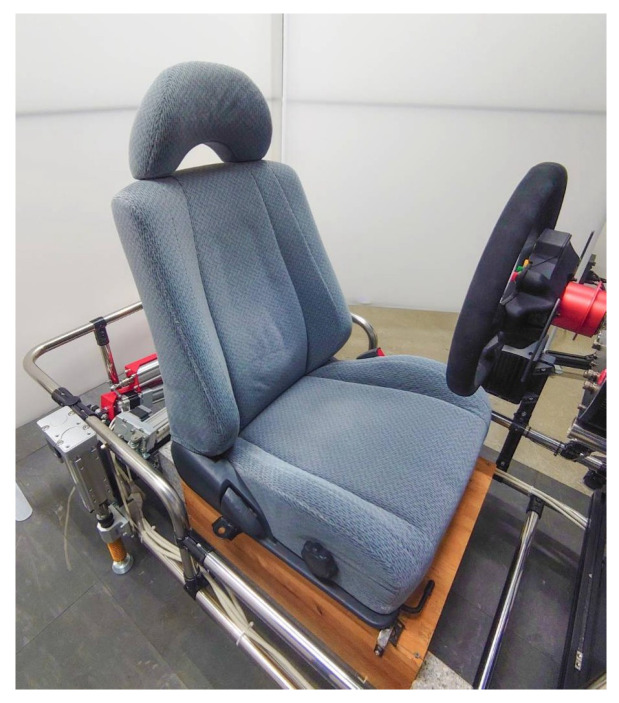
The seat of the driving simulator.

To reproduce the sensation of real driving, the vehicle dynamics and steering feedback were optimized, and the driving environment simulated a clear midnight condition. The seat of the driving simulator is shown in Figure 3.

To promote drowsiness, the seat was a relatively soft fabric seat originally used in an actual vehicle.

### 3.2. Drowsiness Annotation by Facial Expression Evaluation

To obtain ground-truth labels of drowsiness, facial expressions were evaluated by raters based on established criteria.

Since a strong correlation has been confirmed between subjective drowsiness ratings and facial expressions, it is possible to evaluate drowsiness based on facial expressions. In this study, following the criteria defined by Kitajima et al. [38] (Table 3), five evaluators other than the subjects assessed the drowsiness levels based on facial expression videos recorded during the experiment. These criteria [38] have been widely applied in subsequent driver monitoring research and incorporated into the national guidelines for driver monitoring systems issued by the Ministry of Land, Infrastructure, Transport and Tourism [4]. Furthermore, the validity of this facial expression-based framework has been empirically supported by recent research. Uchiyama et al. [39] demonstrated that the observer rating of drowsiness (ORD) based on the Kitajima et al. (1997) criteria [38] showed significant correlations with subjective (Karolinska Sleepiness Scale), physiological (EEG θ/α ratio), and behavioral (driving performance) indices, confirming its convergent validity. Therefore, the present labeling approach is consistent with established and validated methodologies for evaluating driver drowsiness.

### 3.3. Dataset

The dataset was constructed from the acquired NIR facial images and the corresponding drowsiness labels, followed by preprocessing for CNN input.

#### 3.3.1. Input Images

**Figure 4:**
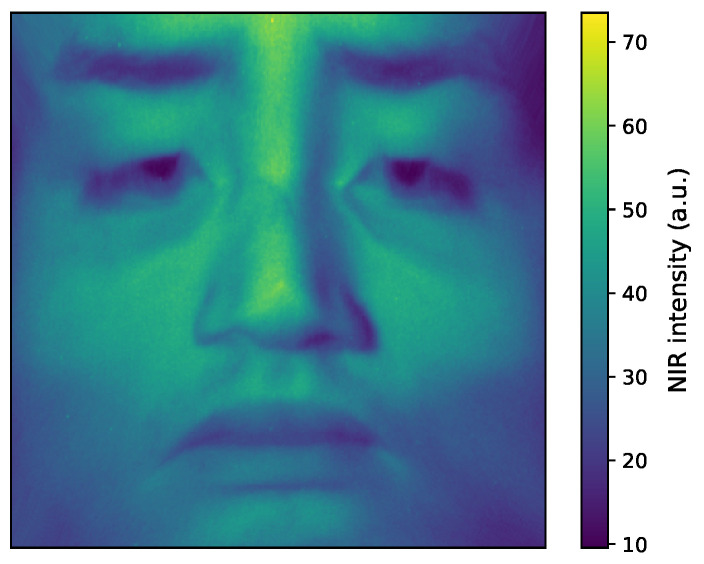
An example of input near-infrared images after spatial normalization.

This subsection describes how facial regions were extracted and standardized to generate the CNN input images. All near-infrared images acquired in the experiment were processed to extract the facial region using the method proposed in the previous study [40]. To further reduce the influence of inter-individual differences in facial orientation and morphology, spatial normalization [41] was applied. Finally, the images were resized to 259×259 pixels for use as input to the CNN. Figure 4 shows an example of input near-infrared images after spatial normalization.

These images were normalized immediately before being fed into the CNN, such that their maximum and minimum values were set to 1 and 0, respectively.

#### 3.3.2. Labels

The labeling scheme for drowsiness levels and the redefinition of classes into three patterns (A, B, C) are summarized here.

Table 4 shows the number of data samples for each subject categorized according to the five-level drowsiness ratings.

In view of the fact that the objective of this study is to capture the early stages of drowsiness and to make use of data from subjects who did not exhibit levels 4 or 5, the drowsiness levels were redefined. The redefined levels are presented in Table 5.

In Pattern A, Level 1 was redefined as Low, and Levels 2 to 5 as High. In Pattern B, Levels 1 and 2 were redefined as Low, and Levels 3 to 5 as High. In Pattern C, Level 1 was redefined as Low, Level 2 as Medium, and Levels 3 to 5 as High. Table 6 shows the number of samples for each subject after redefining the levels (patterns A, B, C).

Furthermore, downsampling was performed to equalize the number of samples across drowsiness levels and among subjects for fair machine learning. The number of samples after downsampling is shown in Table 7.

### 3.4. CNN Modeling

The architecture and configuration of the convolutional neural network (CNN) used for drowsiness classification are detailed in this subsection.

The architecture of the CNN is summarized in Table 8.

Here, Conv, MaxPool, Dropout, and FC denote a convolutional layer, max-pooling layer, dropout layer, and fully connected layer, respectively. To ensure comparability with our previous study (currently under review), all hyperparameters—including the number of units per layer, network depth, learning rate, number of convolutional filters, kernel size, pooling window, batch size, and dropout rate—were kept identical to those used in that work. As a result, the CNN was configured with five convolutional layers, four max-pooling layers, two dropout layers, and two fully connected layers; Dropout1 and Dropout2 disabled 25% and 50% of the connections, respectively. The network was trained using the RMSprop optimizer with a learning rate of 0.001. The loss function was categorical cross-entropy, and training was performed for 20 epochs with a batch size of 30. Early stopping was not applied in this study, and a fixed random seed of 1 was used to ensure reproducibility. No data augmentation techniques were applied in this pilot study. As the pixel intensity of NIR images directly reflects physiological absorption characteristics, conventional geometric or photometric data augmentation was not applied to avoid introducing non-biological artifacts.

### 3.5. Model Evaluation Method

Model performance was evaluated using leave-one-subject-out cross-validation to assess generalizability across individuals.

To evaluate model performance, a 10-fold leave-one-subject-out cross-validation (LOSO CV) was carried out. For each fold, images from one subject were withheld for testing, while the remaining subjects provided the training data. The accuracies obtained from all folds were averaged to determine the final overall accuracy. This analysis was conducted independently for three label assignment strategies: Pattern A, Pattern B, and Pattern C.

### 3.6. Grad-CAM

Grad-CAM was employed to visualize the discriminative facial regions that contributed to the CNN’s classification decisions.

Grad-CAM [36] was employed to visualize the regions that contributed to CNN decisions. It was computed using the trained models and test images, and only correctly classified samples from each subject (i.e., those in which the predicted and annotated drowsiness levels matched) were analyzed. For each class and subject, the Grad-CAM maps ($L_{Grad-CAM}^{c}$) were averaged. Regions with higher average Grad-CAM values indicate the areas utilized by the CNN when determining the drowsiness level. In this study, Grad-CAM was applied exclusively to the classification pattern that achieved the highest accuracy. We present the averaged Grad-CAM maps superimposed on representative facial near-infrared images for each subject.

## 4. Results

### 4.1. CNN Performance

**Figure 5:**
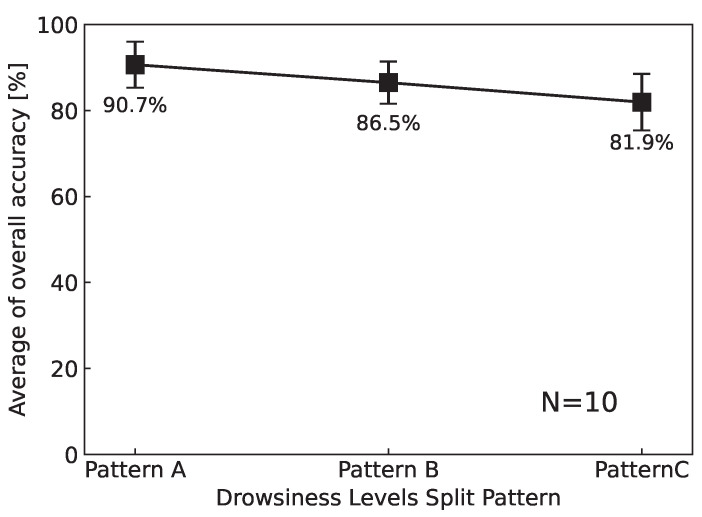
Average of overall accuracy (%) (N = 10). Error bars represent the standard error during 10-fold cross-validation.

Figure 5 presents the average overall classification accuracy of the CNN model for the three label assignment strategies (Patterns A, B, and C).

The results are shown as the mean accuracy across all ten subjects, with error bars indicating the standard error obtained from 10-fold leave-one-subject-out cross-validation (LOSO CV). As seen in the figure, the highest classification performance was achieved with Pattern A, yielding an average accuracy of approximately 90%. This pattern, in which Level 1 was defined as “Low” and Levels 2–5 as “High,” provided the clearest separation of drowsiness states and demonstrated the most robust generalization across individuals.

In contrast, Pattern B, which grouped Levels 1–2 as “Low” and Levels 3–5 as “High,” exhibited a slight reduction in performance, with an average accuracy of approximately 85%. This decrease suggests that merging Level 2 into the “Low” group increased the intra-class variability, thereby complicating the boundary between drowsiness and non-drowsiness states.

Pattern C, which divided the data into three categories (Low: Level 1, Medium: Level 2, High: Levels 3–5), showed the lowest performance, with an average accuracy of approximately 82%. The increased difficulty of distinguishing between three drowsiness levels, especially in separating the “Medium” category from its adjacent levels, is considered a primary factor in this decline. Furthermore, because Pattern C involved downsampling to balance three classes, the reduction in training data may have also contributed to the lower accuracy compared to binary classification strategies.

Overall, these results demonstrate that the binary classification framework, particularly Pattern A, is most effective for CNN-based drowsiness detection from facial near-infrared images. Therefore, subsequent Grad-CAM analysis in this study was conducted using Pattern A, which achieved the highest overall classification accuracy.

In addition to the overall trends shown in Figure 5, the subject-wise classification accuracies are summarized in Table 9.

These results reveal that Pattern A not only achieved the highest mean accuracy but also demonstrated stable performance across individuals, exceeding 90% in eight out of ten subjects. By contrast, Patterns B and C showed greater inter-subject variability, with certain cases exhibiting relatively low accuracies (e.g., SubB: 66.0% in Pattern B; SubA: 45.2% in Pattern C). This variability highlights the challenge of achieving reliable generalization across subjects, particularly when using more complex label assignment strategies. Nevertheless, the consistently strong performance of Pattern A across most participants reinforces its suitability as the most effective framework for NIR-based drowsiness classification.

### 4.2. Average Grad-CAM Activation Map

**Figure 6:**
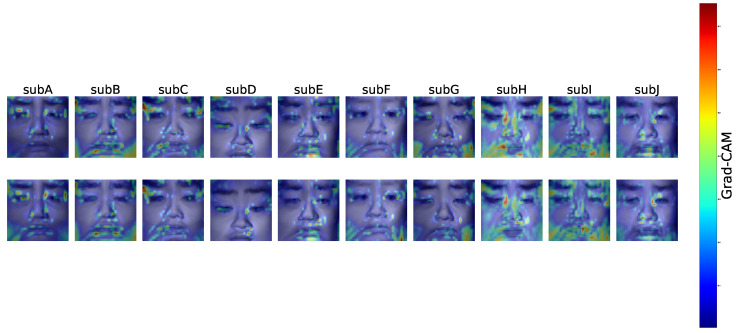
Average Grad-CAM activation maps (Pattern A). Higher activations were found primarily in the periocular region, with additional responses along the nasal dorsum, perioral area, and forehead.

To elucidate the decision-making process of the CNN, Grad-CAM analysis was conducted using Pattern A, which achieved the highest classification accuracy. Figure 6 presents the averaged Grad-CAM heatmaps computed from correctly classified samples of each subject.

The regions displayed in warm colors indicate the areas that contributed most strongly to the CNN’s prediction of drowsiness levels.

Overall, the CNN did not distribute its attention uniformly across the entire face but instead concentrated on physiologically meaningful regions. A notable finding common to all participants was the consistent activation in the periocular area. In subjects with relatively higher classification accuracy (e.g., SubE, SubH, SubI, SubJ), additional activations were also observed along the nasal dorsum and in the perioral region. While periocular activations were most prominent, noticeable responses were likewise observed in the nose and forehead. These regions are associated with variations in skin blood flow and hue [26,42,43,44], suggesting that subtle physiological changes related to drowsiness were likely captured in the near-infrared images. Nevertheless, it should also be considered that activations in the periocular region may, at least in part, reflect behavioral cues such as blinking or eyelid closure.

## 5. Discussion

This study investigated the feasibility of drowsiness classification based on facial near-infrared (NIR) images using a convolutional neural network (CNN). The results demonstrated that Pattern A, which defined Level 1 as “Low” and Levels 2–5 as “High,” achieved the highest classification accuracy of approximately 90%. Grad-CAM analysis further confirmed that the CNN focused on physiologically meaningful regions, particularly around the eyes and the nasal dorsum, suggesting that the model captured facial features closely associated with drowsiness. Subject-wise accuracies also indicated consistently high performance across individuals, despite some inter-individual differences in the extent of activation regions. These findings collectively suggest that CNN-based analysis of NIR facial images can serve as a promising approach for detecting drowsiness.

Compared to conventional commercial driver-monitoring systems (DMS), which primarily rely on behavioral cues such as eyelid closure, blink rate, or head movement, the present approach offers several advantages. Behavioral features often require temporal accumulation of data and are typically detected only after drowsiness has already advanced. By contrast, our results demonstrate that NIR facial images can be treated as physiological indicators that reflect subtle changes associated with drowsiness, enabling earlier detection. In particular, the structure of Pattern A, which distinguished a completely alert state (Level 1) from all other states (Levels 2–5), highlights its potential applicability for identifying the early onset of drowsiness—a capability that conventional behavior-based methods struggle to achieve.

In addition to conventional behavior-based methods, drowsiness estimation from NIR facial images has previously been attempted using sparse coding [20]. While sparse coding requires handcrafted feature extraction and may be constrained in capturing complex spatial representations, the present CNN-based approach learns features in an end-to-end manner, offering greater flexibility and adaptability. Although the current pilot study is limited by sample size, this comparison suggests that CNNs may provide a more robust framework for leveraging NIR facial images in drowsiness detection.

Despite these encouraging results, several limitations must be acknowledged. First, the classification accuracy of 90% is still insufficient for practical deployment in safety-critical contexts such as driving. Even a small number of misclassifications could lead to false alarms or missed detections, which would directly affect driver trust and road safety. Second, the experimental conditions were strictly controlled: participants were required to sleep for more than six hours prior to the experiment, abstain from eating or drinking (other than water), smoking, sleeping, and exercising for two hours before the experiment, and participate without makeup, sunscreen, or glasses (except contact lenses). These conditions may not reflect realistic driving environments, where external factors such as lighting, fatigue history, and personal habits vary considerably. Although the present experiment was conducted under controlled conditions to minimize external noise, previous research has demonstrated that physiological responses related to driver drowsiness in simulator environments are comparable to those in real-world driving [45,46,47]. Furthermore, our group recently developed an artifact correction method that compensates for environmental temperature and time-of-day variations in facial thermography [48], demonstrating that facial physiological imaging can be adapted for use in uncontrolled environments. These findings collectively suggest that the proposed NIR-based approach has practical potential for real-world driver monitoring applications.

Third, the drowsiness labels in this study were assigned based on third-party evaluations of facial expressions. Although no physiological gold standard (e.g., EEG or ECG) or subjective ratings was used for annotation, inter-rater reliability analysis demonstrated acceptable consistency among the five evaluators. Specifically, the intraclass correlation coefficient indicated good agreement across raters (ICC(2,k) = 0.92), while Fleiss’ κ showed fair exact agreement (κ=0.29), reflecting minor categorical variations that commonly occur in behavioral annotation.

Fourth, the CNN architecture and hyperparameters were not optimized for this task, leaving considerable room for further improvement.

It should be noted that this pilot study did not perform direct temporal validation or comparison with behavioral-based indicators. Our statement on “earlier detection” should therefore be regarded as a potential advantage of the proposed single-frame NIR approach, rather than as a result directly validated by comparison. Future work will include explicit temporal analyses and direct comparisons with conventional behavior-based systems to rigorously confirm this capability.

Future research should address these limitations by validating the approach under real driving conditions, incorporating a larger and more diverse subject pool that includes participants from different age groups and ethnic backgrounds, and introducing multimodal labels that combine subjective scales and physiological measurements with external ratings. In particular, future studies will include validation using physiological reference signals such as electroencephalography (EEG) and electrocardiography (ECG) to clarify the physiological relevance of NIR intensity and improve the robustness of the labeling process. In addition, further optimization of the CNN model, including architecture refinement, data augmentation, and parameter tuning, is expected to enhance classification performance. Integration with conventional behavioral indicators may also lead to a hybrid driver-monitoring system that combines the strengths of both physiological and behavioral monitoring. Finally, systematic evaluations of algorithm performance, vulnerability testing, and comparisons with existing state-of-the-art drowsiness detection methods will be necessary to demonstrate the practical advantages of the proposed framework.

In summary, this pilot study demonstrated that CNN-based analysis of facial NIR images can classify drowsiness with high accuracy and interpretability. While the current performance is not yet sufficient for practical application, the approach provides a foundation for developing advanced driver-monitoring technologies that enable earlier and more reliable detection of drowsiness than conventional systems.

From a practical standpoint, the results suggest that NIR-based CNN analysis could complement existing behavior-based DMS by enabling earlier and potentially more reliable drowsiness detection. In particular, Pattern A, which distinguishes an awake state (Level 1) from all other states (Levels 2–5), demonstrates potential applicability for early warning systems. This framework may be compatible with commercial in-cabin cameras that already employ NIR illumination at 940 nm, thereby enhancing real-time monitoring without requiring substantial hardware modifications. Ultimately, such capability could support safer transitions in semi-automated driving by allowing timely interventions before severe drowsiness occurs.

## 6. Conclusions

This pilot study explored the feasibility of classifying driver drowsiness using facial near-infrared (NIR) images and a convolutional neural network (CNN). By employing the 940 nm wavelength band already used in commercially available driver-monitoring cameras, we demonstrated that physiological features embedded in NIR facial images can serve as effective indicators of drowsiness. Among the three label assignment strategies, the binary classification scheme (Pattern A), which defined Level 1 as “alert” and Levels 2–5 as “drowsy,” achieved the highest average accuracy of approximately 90%. Although this level of accuracy is not yet sufficient for direct practical implementation, the results suggest that the CNN successfully captured physiological cues associated with drowsiness. Importantly, because Pattern A distinguishes between an awake state and all other levels, it shows potential for supporting early detection of drowsiness.

Furthermore, Grad-CAM analysis revealed that the CNN focused on physiologically meaningful regions such as the periocular, nasal dorsum, perioral, and forehead when making classification decisions. These regions correspond well with known drowsiness-related changes, supporting the reliability of the model. The analysis also showed inter-individual variations in highlighted areas, indicating that the CNN flexibly adapted to individual facial characteristics while consistently attending to relevant regions across subjects.

Nevertheless, several limitations should be acknowledged. The drowsiness labels were derived from third-party evaluations of facial expressions, without incorporating subjective self-reports or physiological references such as EEG or ECG. In addition, no extensive model optimization was conducted in this study, suggesting room for further improvement in classification accuracy and generalization. Future work should address these issues by integrating multimodal data, increasing subject diversity, and applying more advanced network architectures.

In summary, this study provides the first evidence that CNN-based analysis of NIR facial images can be leveraged as a physiological approach for driver drowsiness detection. While challenges remain for practical application, NIR imaging holds promise as a means for achieving earlier and more reliable detection of drowsiness compared to conventional behavior-based systems.

## Figures and Tables

**Table 1:** Representative studies on driver monitoring based on behavioral and physiological indicators.

Main Indicator/Input	Model/Method	Key Findings/ Characteristics	Main Limitation
Blink kinematics [18]	Statistical/regression analysis	Blink parameters associated with degraded driving performance after sleep deprivation	Mainly detects advanced drowsiness; early sensitivity limited.
Behavioral indicators (mirror checks, head/body posture) [17]	Statistical feature analysis	Behavioral indicators such as mirror-check frequency linked to drowsiness progression(No significant difference)	Weak changes in early drowsiness stage.
Facial landmarks (68 points) [13]	Deep Learning	Classified driver behaviors from facial landmark dynamics using infrared facial images	Sequence dependence; limited instantaneous response.
Behavioral indicators [12]	Simulation dummy design/system evaluation	Developed a driver-monitoring evaluation dummy and validated DMS performance under fatigue scenarios	Hardware-oriented validation; not a direct learning model.
Temporal gaze variation [16]	On-road experiment with multimodal alerts	Investigated gaze behavior and visual attention during distraction countermeasures	Not targeted at subtle or short-term drowsiness.
EEG power ratios (θ/α etc.) [36]	Deep Learning	Demonstrated deep-learning-based vigilance estimation from EEG signals under sleep deprivation	Requires electrode attachment; noise-sensitive.
Prefrontal hemoglobin changes [37]	LDA/SVM	Hybrid EEG–fNIRS framework; reported vigilance/drowsiness-related cerebral hemodynamic changes	Bulky device; long temporal response.

**Table 2:** Experimental protocol and durations of each phase.

Phase	Duration	Eye State
Rest 1	1 min	Eyes open
	1 min	Eyes closed
	1 min	Eyes open
Driving	40 min	Not controlled
Rest 2	1 min	Eyes open
	1 min	Eyes closed
	1 min	Eyes open

**Table 3:** Drowsiness rating criteria based on facial expression by the drowsiness assessment method [38].

Drowsiness Level	State	Example of Facial Expression and Motions
Level 1	Awake	· Eye movements are quick and frequent
		· Blink cycles are stable at approximately two per two seconds
		· Body motions are active
Level 2	Slightly drowsy	· Lips are parted
		· Motions of eye movements are slow
Level 3	Drowsy	· Blinks are slow and frequent
		· Re-positions body on seat
		· Touches hand to face
Level 4	Very drowsy	· Blinks assumed to occur consciously
		· Shakes head
		· Frequently yawns
Level 5	Extremely drowsy	· Eyelids closing
		· Leans head back and forth

**Table 4:** Number of samples for each subject by drowsiness level (Original 5-level ratings [38]).

Subject	Level 1	Level 2	Level 3	Level 4	Level 5
SubA	440	880	680	400	0
SubB	480	280	560	1080	0
SubC	340	1580	480	0	0
SubD	640	620	1140	0	0
SubE	320	280	880	920	0
SubF	480	1040	460	360	60
SubG	580	760	1060	0	0
SubH	320	200	400	1160	320
SubI	340	560	720	760	20
SubJ	320	180	180	1480	240

**Table 5:** Redefinition of levels in each pattern.

Drowsiness Level	Pattern A	Pattern B	Pattern C
Level 1	Low	Low	Low
Level 2	High	Low	Medium
Level 3	High	High	High
Level 4	High	High	High
Level 5	High	High	High

**Table 6:** Number of samples for each subject after redefinition of levels (Patterns A, B, C).

	Pattern A		Pattern B		Pattern C		
Subject	Low	High	Low	High	Low	Medium	High
SubA	440	1960	1320	1080	440	880	1080
SubB	480	1920	760	1640	480	280	1640
SubC	340	2060	1920	480	340	1580	480
SubD	640	1760	1260	1140	640	620	1140
SubE	320	2080	600	1800	320	280	1800
SubF	480	1920	1520	880	480	1040	880
SubG	580	1820	1340	1060	580	760	1060
SubH	320	2080	520	1880	320	200	1880
SubI	340	2060	900	1500	340	560	1500
SubJ	320	2080	500	1900	320	180	1900
Total	4260	19,740	10,640	13,360	4260	6380	13,360

**Table 7:** Number of samples for each subject after downsampling to the within-pattern class minimum (Patterns A, B, C).

	Pattern A		Pattern B		Pattern C		
Subject	Low	High	Low	High	Low	Medium	High
SubA	320	320	480	480	180	180	180
SubB	320	320	480	480	180	180	180
SubC	320	320	480	480	180	180	180
SubD	320	320	480	480	180	180	180
SubE	320	320	480	480	180	180	180
SubF	320	320	480	480	180	180	180
SubG	320	320	480	480	180	180	180
SubH	320	320	480	480	180	180	180
SubI	320	320	480	480	180	180	180
SubJ	320	320	480	480	180	180	180
Total	3200	3200	4800	4800	1800	1800	1800

Note. Within each pattern, all classes were downsampled to the minimum class count observed in that pattern: Pattern A → 320; Pattern B → 480; Pattern C → 180.

**Table 8:** Construction of CNN. Conv, MaxPool, Dropout, and FC indicate a convolutional layer, max pooling layer, dropout layer, and fully connected layer, respectively.

Layer	Output Shape	Activation	Parameters
Conv1	(259, 259, 32)	ReLU	320
Conv2	(257, 257, 64)	ReLU	18,496
MaxPool1	(128, 128, 64)	–	0
Conv3	(126, 126, 64)	ReLU	36,928
MaxPool2	(63, 63, 64)	–	0
Conv4	(61, 61, 64)	ReLU	36,928
MaxPool3	(30, 30, 64)	–	0
Conv5	(28, 28, 64)	ReLU	36,928
MaxPool4	(14, 14, 64)	–	0
Dropout1	(14, 14, 64)	–	0
Flatten	(12,544)	–	0
FC1	(128)	ReLU	1,605,760
Dropout2	(128)	–	0
FC2 (Output)	(2 or 3)	Softmax	258
Total			1,735,618

**Table 9:** Subject-wise classification accuracy (%) across three label assignment patterns (10-fold LOSO CV). Mean ± SD and Median are computed across subjects.

Subject	Pattern A	Pattern B	Pattern C
SubA	47.3	64.0	45.2
SubB	76.1	66.0	55.7
SubC	92.5	63.3	59.1
SubD	93.4	89.1	80.4
SubE	98.1	97.7	97.4
SubF	99.8	93.2	89.8
SubG	99.7	95.2	97.0
SubH	99.8	99.0	99.1
SubI	99.7	97.5	97.0
SubJ	100.0	99.8	98.7
Mean ± SD	90.7 ± 16.9	86.5 ± 15.5	81.9 ± 20.8
Median	98.9	94.2	93.4

## Data Availability

The data that support the findings of this study are available from the corresponding author upon reasonable request.
